# Biomarker analysis in human neoplasias: superior next-generation sequencing on frozen bone marrow cells and on formalin-fixed, paraffin-embedded tumor tissues

**DOI:** 10.1186/1753-6561-7-S2-K18

**Published:** 2013-04-04

**Authors:** Sakari Knuutila

**Affiliations:** 1 Department of Pathology and Genetics, Haartman Institute, Helsinki University and HUSLAB, Finland

## Background

Biomarker-based personalized treatment has improved the prognosis of many tumors. *EGFR*, *KRAS*, and *BRAF* mutations and EGFR antibody treatment (colorectal carcinoma) and EGFR inhibitor treatment (lung carcinoma) serve as good examples. The rapid improvement of biomarker-based treatments requires that not merely one biomarker but many biomarkers such as mutations, gene fusion, and gene copy number alterations, are to be tested simultaneously for selection of the ideal combination of molecularly targeted and individual treatment.

Next-generation sequencing (NGS) makes it possible to study mutations, gene fusions, and copy numbers genome wide in one single analysis. This presentation based on our three papers [1-3] in press illustrates the feasibility, even superiority of NGS for biomarker analysis in human neoplasias.

## Aims

1) As *EGFR*, *KRAS*, and *BRAF* mutations are clinically relevant, mainly predictive biomarkers in colorectal and lung carcinomas, we aimed to evaluate by NGS and routinely used real-time PCR (RT-PCR) methods the feasibility of NGS for analysis of these mutations. Furthermore, we aimed, by NGS, to reveal novel mutations.

2) Anaplastic lymphoma kinase (*ALK*) gene fusions occur in a small proportion of non-small cell lung carcinomas. Identification of the fusions is crucial for targeted treatment decisions. We aimed to screen *ALK* gene fusions in lung carcinomas, and to compare the results that were studied by NGS with those detected by routine methods, including standard fluorescence *in situ* -hybridization (FISH), immunohistochemistry (IHC), and RT-PCR. We conducted the *ALK* fusion analyses on 95 formalin-fixed paraffin-embedded (FFPE) tumor tissues from 87 patients with non-small cell lung carcinoma.

3) In many human neoplasias such as acute lymphoblastic leukemia, chromosomal deletions at 9p are common. To study the feasibility of NGS for detection of copy number alterations at chromosome 9p, we conducted NGS and array-comparative genomic hybridization (aCGH) analyses on 35 patients with acute lymphoblastic leukemia.

## Methods

Methods for NGS, aCGH, FISH, RT-PCR, and IHC appear in the original articles [1-3]. Our NGS method was based on targeted resequencing. For *EGFR*, *KRAS*, *BRAF*, and *ALK*, all the exons (for *ALK* and also some intronic areas) were sequenced. For 9p, target regions were sequenced with a total length of 1 Mb from a 32 Mb region. For capture of target regions, baits were custom-designed to capture all exons.

## Results and discussion

### Comparison of NGS and PCR methods for detection of *EGFR*, *KRAS*, and *BRAF* mutations in lung carcinoma [1]

We observed a significant concordance (from 96.3 to 100%) of the *EGFR*, *KRAS*, and *BRAF* mutation detection results between targeted NGS and RT-PCR. Moreover, targeted NGS revealed seven non-synonymous single-nucleotide variations and one insertion-deletion variation in *EGFR* undetectable by the real-time PCR methods. The potential clinical significance of these variants requires elucidation in future studies. Our results support the use of targeted NGS in the screening of *EGFR*, *KRAS*, and *BRAF* mutations in FFPE tissue material.

### Detection of *ALK* fusions in lung carcinoma by NGS, FISH, RT-PCR, and ICH [2]

All methods were successful on FFPE tumor tissue material. Of the 87 patients examined, we detected *ALK* fusion in 5 (5.7%). Results from resequencing correlated significantly with those from FISH, RT-PCR, and IHC. Targeted NGS proved a promising method for *ALK* gene fusion detection in non-small-cell lung carcinomas. Means to reduce the material and turnaround time required for analysis are, however, necessary.

### NGS as a method for detection of copy number alterations [3]

In detection of copy number alterations at 9p, high concordance occurred between NGS and aCGH. Both methods revealed deletions at the *CDKN2A/B* locus, whereas NGS revealed additional small deletions outside the resolution of aCGH. Furthermore, NGS showed mutations and gene fusions impossible to see by aCGH. To conclude, novel copy number alterations, mutations, and structural alterations were revealed.

## Conclusions

For two reasons, NGS was superior for biomarker analysis: the possibility to see a large range of gene mutations in one analysis, and the possibility to detect mutations, gene fusions, and copy number alterations simultaneously in a single test. Our studies illustrated the feasibility of NGS for clinical specimens of FFPE tumors .We also showed that in addition to mutations, gene fusions and copy number alterations can also be reliably detected by NGS. As the costs and required material for NGS analyses has decreased to near that of PCR or FISH, NGS may very soon become a method to replace all other methods for biomarker analyses of human malignancies.

## Competing interests

There are no competing interests in this presentation.
